# Using *Wolbachia* to control rice planthopper populations: progress and challenges

**DOI:** 10.3389/fmicb.2023.1244239

**Published:** 2023-09-14

**Authors:** Yan Guo, Jiayi Shao, Yanxian Wu, Yifeng Li

**Affiliations:** Guangdong Provincial Key Laboratory of High Technology for Plant Protection, Institute of Plant Protection, Guangdong Academy of Agricultural Sciences, Key Laboratory of Green Prevention and Control on Fruits and Vegetables in South China Ministry of Agriculture and Rural Affairs, Guangzhou, China

**Keywords:** *Wolbachia*, mosquitoes, planthoppers, transmission, cytoplasmic incompatibility, pathogen inhibition

## Abstract

*Wolbachia* have been developed as a tool for protecting humans from mosquito populations and mosquito-borne diseases. The success of using *Wolbachia* relies on the facts that *Wolbachia* are maternally transmitted and that *Wolbachia*-induced cytoplasmic incompatibility provides a selective advantage to infected over uninfected females, ensuring that *Wolbachia* rapidly spread through the target pest population. Most transinfected *Wolbachia* exhibit a strong antiviral response in novel hosts, thus making it an extremely efficient technique. Although *Wolbachia* has only been used to control mosquitoes so far, great progress has been made in developing *Wolbachia*-based approaches to protect plants from rice pests and their associated diseases. Here, we synthesize the current knowledge about the important phenotypic effects of *Wolbachia* used to control mosquito populations and the literature on the interactions between *Wolbachia* and rice pest planthoppers. Our aim is to link findings from *Wolbachia*-mediated mosquito control programs to possible applications in planthoppers.

## Introduction

*Wolbachia* are a group of gram-negative bacteria that live inside invertebrate cells and have been successfully developed to control mosquitoes and mosquito-borne diseases by decreasing host population density or decreasing host virus transmission. Unlike chemical control approaches, which result in collateral destruction of beneficial insects, *Wolbachia*-mediated population control has proven to be an excellent vector-control agent because it targets a single species. Moreover, as the target population is suppressed, the chemical control approaches become less effective, while *Wolbachia*-mediated pest population control is more effective. Because of *Wolbachia* pervasiveness in nature and lack of genetic modification, *Wolbachia*-mediated control programs are accepted as environmentally friendly biocontrol strategies to control insect pest populations and disease vectors. To date, the *Wolbachia* control strategies successfully used have been limited to mosquitoes. There is a question of whether *Wolbachia* control strategies could be applied more broadly to other pest insects and insect-borne diseases.

Rice (*Oryza sativa*), cultivated extensively in the tropical and subtropical regions of the world, is the staple food for billions of people worldwide (Sarao et al., 2016). Rice planthoppers (Hemiptera: Delphacidae), the most destructive pests of rice, suck rice sap and oviposit in rice tissues, inducing a substantial threat to rice production. In addition to heavy infestations, rice planthoppers also act as vectors of major plant viruses, such as rice stripe virus, rice black-streaked dwarf virus, rugged stunt virus, grassy stunt virus, and southern rice black-streaked dwarf virus (Hibino, 1996). Various strategies have been developed to control planthoppers. Among those strategies, spraying chemical insecticides is the main method used for controlling this pest. However, blanket application of insecticides has already induced planthopper resistance and disrupted the ecological balance of rice ecosystems in most rice planting countries. Thus, a more practical, economical and environmentally friendly strategy is urgently needed to control planthoppers and their associated diseases.

The success of *Wolbachia*-mediated mosquito control programs promotes similar strategies that could be applied to planthoppers. Here, we summarize the important properties of *Wolbachia* used for mosquito control, including stability transmission, host reproduction alteration, and pathogen inhibition. We also review the current knowledge about the interactions between *Wolbachia* and planthoppers and point out the similarities and differences in biology between mosquitoes and planthoppers to link findings from *Wolbachia*-mediated mosquito control programs to possible applications in planthoppers.

## *Wolbachia* phenotypes

### *Wolbachia* diversity

*Wolbachia* strains were first identified in the reproductive tissue of *Culex pipiens* in 1924 (Hertig and Wolbach, 1924). Since then, these bacteria have been found to infect approximately half of all arthropod species from terrestrial and aquatic environments, including nematodes, mites, spiders and all orders of insects (Weinert et al., 2015). *Wolbachia* formed a monophyletic group with other insect-associated microorganisms using 16S rRNA gene sequences. In recent decades, a large number of *Wolbachia* with close phylogenetic affinity have been revealed by PCR and sequencing techniques. Based on the variable gene *ftsZ*, *Wolbachia* from arthropods form two divergent clades; several different *Wolbachia strains* from filarial nematodes are assigned to two additional clades (Werren et al., 1995; Bandi et al., 1998). These clades have since been termed supergroups, which are used to describe the divergence of the *Wolbachia* group. In addition, *Wolbachia* surface protein (*wsp*) and *groEL* genes are used to distinguish the major phylogenetic subdivisions of *Wolbachia*. Due to extensive recombination and strong diversifying selection in the *wsp* gene, *wsp* should therefore be unsuitable for use alone for reliable *Wolbachia* strain characterization when trying to type and quantify strain diversity (Werren and Bartos, 2001; Baldo et al., 2005; Lo et al., 2007). Considering that a single-locus approach to strain characterization may be misleading, a multilocus sequence typing (MLST) system has been established to type *Wolbachia* strains using five standard housekeeping genes (*gatB, coxA, hcpA, fbpA, and ftsZ*) (Baldo et al., 2006). Based on the combination of alleles at a sample of housekeeping genes, the MLST approach defines a strain as a sequence type. This accurate strain typing system MLST using combinations of alleles as molecular markers to genotype strains is considered a universal and unambiguous tool for *Wolbachia* strain typing, molecular evolutionary, and population genetics studies (Baldo et al., 2006). Overall, the MLST system provides an excellent method for typing *Wolbachia* strains from diverse hosts and for discriminating among strains in the same host species (Baldo et al., 2006).

**Figure 1:**
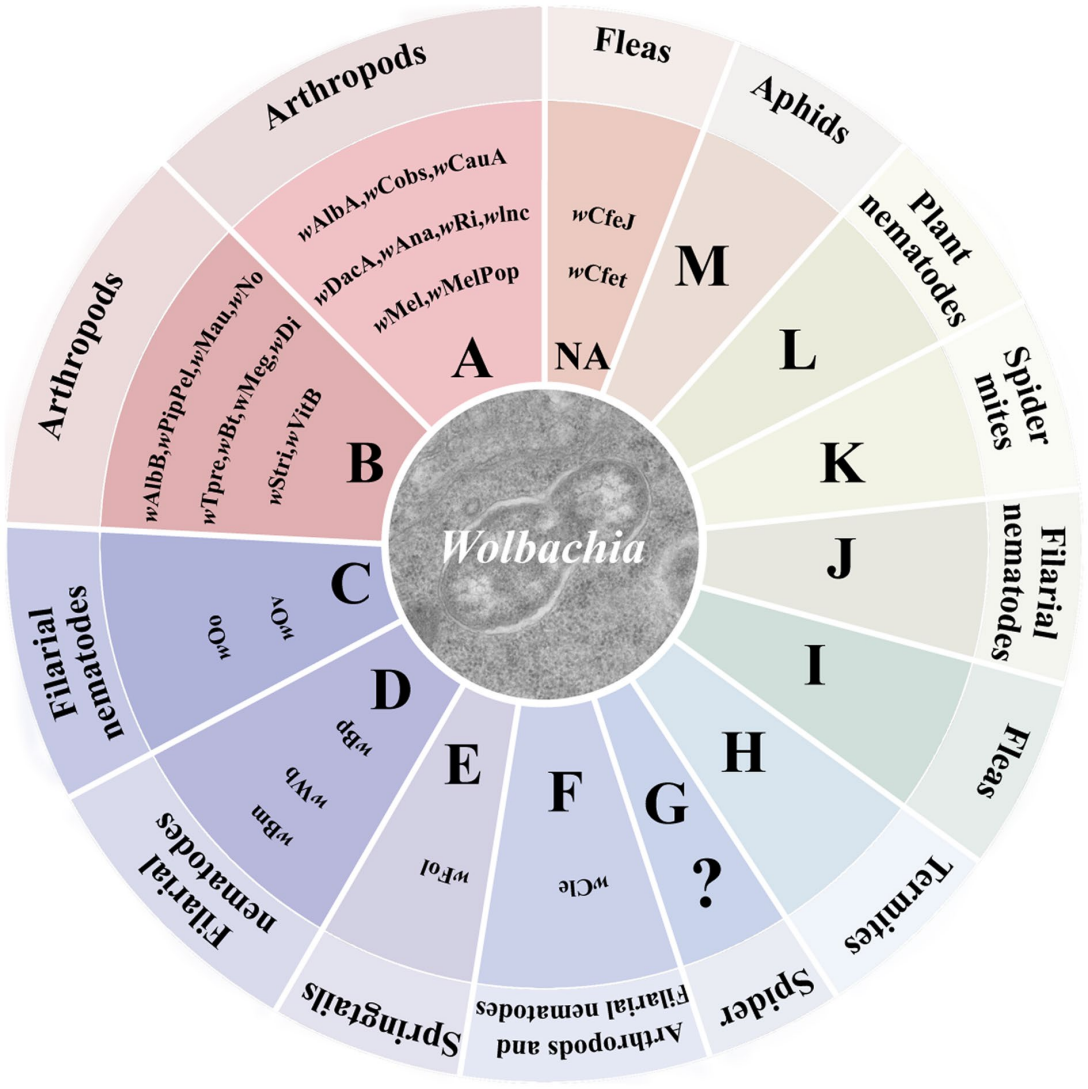
*Wolbachia* supergroups. *Wolbachia* strains are subdivided into different supergroups. Most *Wolbachia* supergroups are listed in the circle graph. Colors correspond to different patterns of *Wolbachia*-host associations across the supergroups. “?”: controversial supergroup; NA, not annotated at supergroup level.

*Wolbachia* strains are subdivided into 17 supergroups from A to R, except for supergroup G, which is controversial (Baldo et al., 2006; Baldo and Werren, 2007; Wang et al., 2016; Zhao et al., 2021). The majority of *Wolbachia* strains found in insects belong to supergroups A and B. Most *Wolbachia* strains that infect arthropods are supergroups A, B, D, E, F, and H (Figure 1). As molecular biology techniques have developed, *Wolbachia* genome sequences are exploited to define genetic diversity and significant genes associated with altering host biology, as well as relationships between *Wolbachia* and hosts at the gene level (Kaur et al., 2021). To date, over 26 complete *Wolbachia* genomes have been published, and nearly 1,000 *Wolbachia* genomes from different arthropod and nematode species have been assembled (Scholz et al., 2020; Kaur et al., 2021). Our understanding of *Wolbachia* genetic diversity is still developing, which will help us to identify useful *Wolbachia* variants with desirable phenotypic effects for alternative *Wolbachia*-mediated population control strategies.

### *Wolbachia* horizontal and vertical transmission

Numerous studies have shown that *Wolbachia* exists in diverse cells and somatic tissues of the host, such as the salivary gland, fat bodies, ovary, testis, midgut, and tegument (Dobson et al., 1999; Toomey et al., 2013). Although *Wolbachia* have been found in host somatic tissues, they exhibit strong reproductive tissue tropism in the host (Frydman et al., 2006; Fast et al., 2011; Toomey et al., 2013). *Wolbachia* are rarely or not transmitted by sperm, while they accumulate in developing spermatocytes of male hosts (Clark et al., 2002; Ijichi et al., 2002; Ju et al., 2017). In female hosts, *Wolbachia* enter ovaries and spread into developing oocytes, eventually dispersing within the offspring of the host (Kose and Karr, 1995; Ferree et al., 2005). Thus, *Wolbachia* is considered as an intracellular maternally transmitted bacterium. The unique ability of *Wolbachia* to invade host populations has rapidly promoted their exploration as a potential tool in the control of pests.

**Figure 2:**
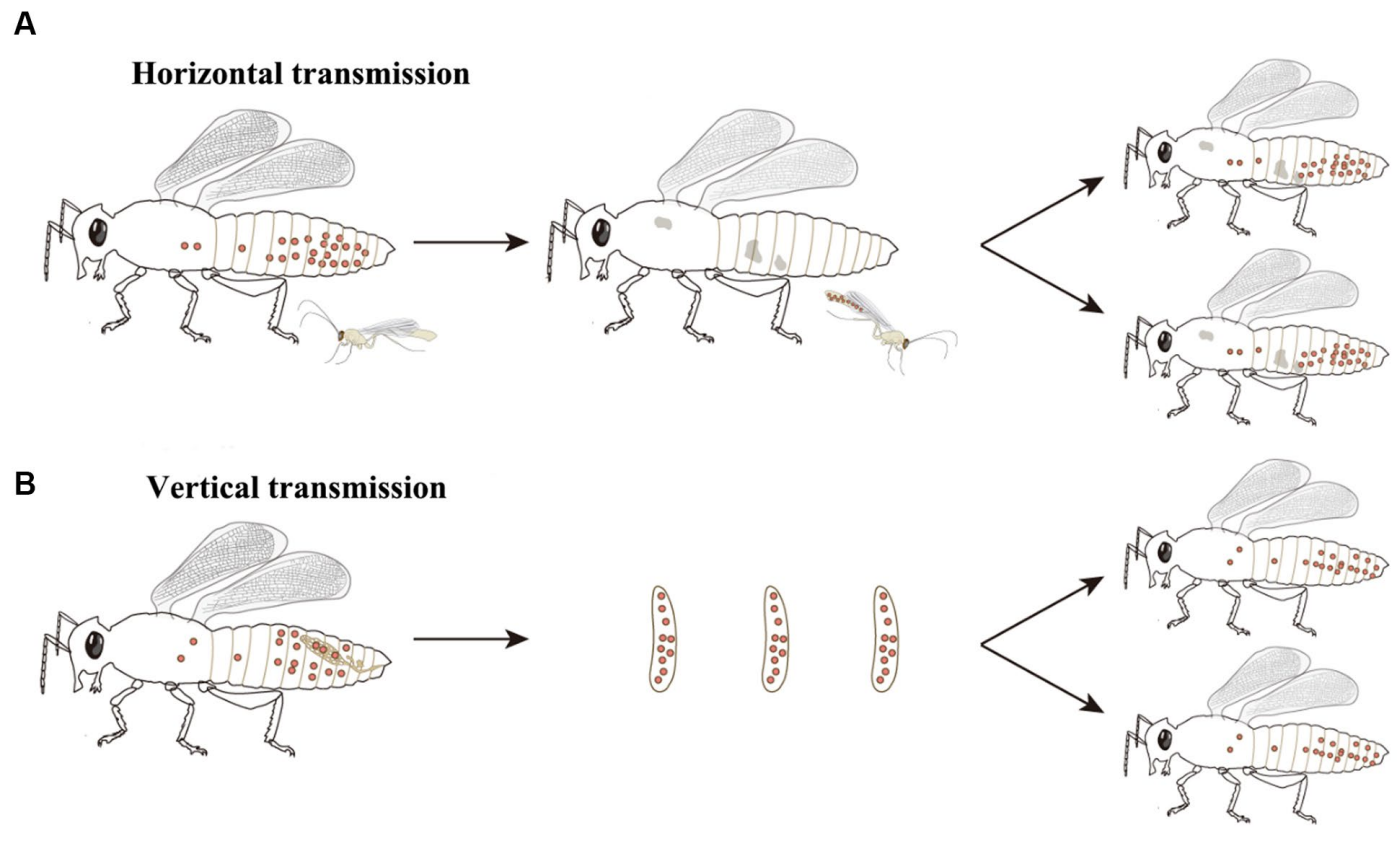
*Wolbachia* transmission. *Wolbachia* persist and disperse in hosts by horizontal and vertical transmission. **(A)** *Wolbachia* horizontally transmit from one species to another. The most common horizontal transmission of *Wolbachia* occurs by parasitic wasps. Parasitic wasps infect *Wolbachia* when they parasitize a *Wolbachia*-infected host, then transfer *Wolbachia* to new hosts when they parasitize closest species. **(B)** *Wolbachia* vertically transmit from mother to offspring. In female hosts, *Wolbachia* infect the germinal tissues, enter into the developing oocytes and be incorporated into the embryos, eventually dispersing within the offspring of host. Red dots: *Wolbachia*.

*Wolbachia* persist and disperse in arthropods and filarial nematodes that mostly depend on their horizontal and vertical transmission. *Wolbachia* can transfer from one species to another, that is, horizontal transmission (Figure 2A), though it has low transmission efficiency. Phylogenetic incongruence between *Wolbachia* and their hosts suggests that horizontal transmission of *Wolbachia* occurs frequently between many hosts (Baldo et al., 2006; Su et al., 2019). MLST analysis of *Wolbachia* and successful horizontal transfer of *Wolbachia* by microinjection have also provided evidence for horizontal transmission (Xi et al., 2005, 2006; Li et al., 2017; Zheng et al., 2019). As recorded, horizontal transmission of *Wolbachia* could occur by many pathways, such as feeding on common plants (Sintupachee et al., 2006; Le Clec'h et al., 2013; Li et al., 2017; Sanaei et al., 2023), parasitic wasps (Ahmed et al., 2015; Brown and Lloyd, 2015; Goya et al., 2022), parasitic mites (Houck et al., 1991; Jaenike et al., 2007; Gehrer and Vorburger, 2012), hybridization (Jiang et al., 2018; Su et al., 2019), and predation (Goodacre et al., 2006; Wang et al., 2010; Su et al., 2019). Although interspecific horizontal transmission inefficiently occurs, *Wolbachia* horizontal transmission is found in many insects, including rice planthoppers (Zhang et al., 2013), wasps (Huigens et al., 2004; Goya et al., 2022; Zhou et al., 2022), fruit flies (Turelli et al., 2018), trypetids (Schuler et al., 2013), psyllids (Serbina et al., 2022), moths (Ahmed et al., 2016), ladybirds (Shaikevich and Romanov, 2023), mosquitoes (Shaikevich et al., 2019), mites (Su et al., 2019), butterflies (Ahmed et al., 2016; Zhao et al., 2021) and so forth.

*Wolbachia* can also vertically transmit from mother to offspring via the host egg cytoplasm (Figure 2B), which is considered the main pathway for infection transfer across hosts (Werren, 1997). Vertical transmission of symbionts in hosts is generally maternal and occurs through trans eggs and transovarial transmission (Rosen, 1988; Lequime et al., 2016). In *trans*-egg transmission, *Wolbachia* spread into eggs at the time of oviposition. In transovarial transmission, *Wolbachia* infect the germinal tissues and enter into the developing oocytes of the female host. When *Wolbachia* initially infect a new host, they need to reach the germinal tissues for successful transovarial transmission (Werren et al., 2008). *Wolbachia* transovarial transmission relies on the infection of developing oocytes, which results in nearly 100% infection of the host progeny (Lequime et al., 2016). Due to the difficultly of detecting trans-egg transmission *in vitro* and vivo, *Wolbachia* vertical transmission in the host is mostly focused on transovarial transmission. The factors that impact *Wolbachia* vertical transmission are complex and undistinguishable and are related to *Wolbachia* densities, interactions with other symbionts, and the ability of *Wolbachia* to migrate into the host oocyte.

*Wolbachia* vertical transmission has been intensively investigated in Diptera insects. *Drosophila* ovarioles are of the polytrophic meroistic type and divide into the terminal filament, germarium, and vitellarium from tip to pedicel (Szklarzewicz et al., 2007; Swiatoniowska et al., 2013; Szklarzewicz et al., 2013). The female germline stem cell niche (GSCN) is on the apical tip of the germarium, where germline stem cells divide asymmetrically, and one daughter cell exits the GSCN and forms the egg’s germline (Fast et al., 2011). Germline cells divide and form egg chambers in the germarium and finally mature into eggs in the vitellarium. Observation research found an intense accumulation of *Wolbachia* in the GSCN and the somatic stem cell niche (SSCN), which is located at the germarium and supports somatic stem cells (Frydman et al., 2006; Fast et al., 2011). Further research showed that *Wolbachia* enter the ovaries of *Drosophila* from the anterior tip of the germarium (Martinez et al., 2014). After that, *Wolbachia* utilize the host actin cytoskeleton during oogenesis for efficient transmission and maintenance between *Drosophila* generations (Newton et al., 2015). Actin-inhibiting drugs significantly abrogate *Wolbachia* uptake in the host, indicating that the host actin cytoskeleton plays an important role in *Wolbachia* transmission (Ferree et al., 2005; Newton et al., 2015; Nevalainen et al., 2023).

### *Wolbachia*-induced cytoplasmic incompatibility

*Wolbachia* impact the ecology, evolution, and reproductive biology of their host species to increase their already widespread distribution. *Wolbachia* are best known for their effects on host reproduction, such as male killing, feminization, thelytokous parthenogenesis, and cytoplasmic incompatibility (CI) (Werren et al., 2008). In 1971, *Wolbachia* were first verified to be associated with CI, causing the embroys of hosts to perish, which occurs when males carrying *Wolbachia* mate with females that are uninfected or harboring different *Wolbachia* strains (Yen and Barr, 1971; Werren, 1997; Hoffmann, 2020). A range of negative fecundity effects or no effects associated with *Wolbachia* has been described, although the mechanisms responsible for those fitness effects are mostly unknown. Other *Wolbachia* strains exhibit strong positive fecundity effects on their host, including fecundity increases (Fast et al., 2011; Guo et al., 2018a).

**Figure 3:**
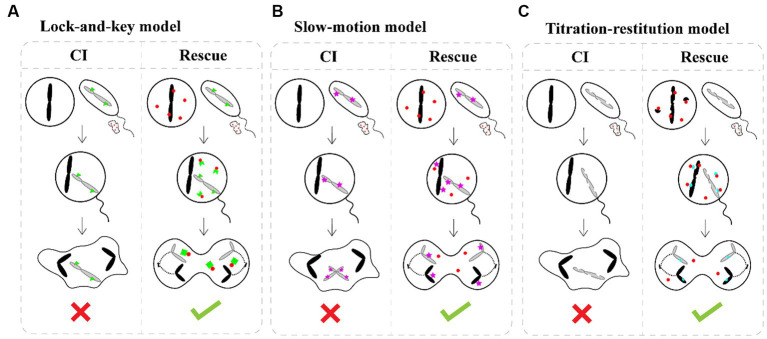
CI induction and rescue models. **(A)** The “lock-and-key” model. *Wolbachia* (red dots) produce a “lock” (green triangle) binding on paternal chromosomes. *Wolbachia* are shed with most of the cytoplasm as spermatogenesis. Cytoplasmic incompatibility occurs in crosses between infected males and uninfected female because the paternal material is “locked-in,” while eggs infected by *Wolbachia* remain compatible after fertilization because *Wolbachia* produce a “key” in the egg which removes the lock. **(B)** The “slow-motion” model. *Wolbachia* (red dots) produce a slowing down factor (purple star) binding on paternal chromosomes. After that, *Wolbachia* are shed from the maturing spermatocyte. Embryonic mortality occurs in crosses between infected males and uninfected females because *Wolbachia* slow down paternal chromosomes movements during the first embryonic mitosis, which is rescued by the similar modification of maternal chromosomes when *Wolbachia* are present in the egg. **(C)** The “titration-restitution” model. *Wolbachia* (red dots) titrate out a protein (semicircles) of paternal and maternal chromosomes. The titrated protein of paternal chromosomes is expelled as *Wolbachia* are shed from the maturing spermatocyte. Cytoplasmic incompatibility occurs when sperm cell enters an uninfected egg due to lack of the host protein. Rescue occurs between two infected individuals, because the *Wolbachia* in eggs give back the host protein (blue semicircles) to maternal and paternal chromosomes.

How *Wolbachia* manipulate the reproduction of hosts, especially *Wolbachia-*induced CI, has attracted great attention in recent decades. Although the means by which *Wolbachia* mediate CI are currently unknown, there is a general consensus that *Wolbachia* modify sperm at an early stage of spermatogenesis, and a rescue activity takes place in the same *Wolbachia*-infected egg to reverse or neutralize the modification of sperm following fertilization (Werren, 1997; Xiao et al., 2021). Three different models have been proposed to account for the mechanisms of CI induction and rescue: the “lock-and-key,” “slow-motion,” and “titration-restitution” models (Figure 3; Poinsot et al., 2003). Moreover, *Wolbachia* genes involved in modification and rescue have been identified, which are collectively named *cifA* and *cifB*. The two genes are organized into an operon-like genetic element, which encodes the CifA and CifB proteins (Beckmann et al., 2017; LePage et al., 2017). To distinguish the CI-inducing modifications and CifA rescues viability, two types of functional models for CI have been proposed. In the host modification models, *Wolbachia* Cifs (CifA and CifB) modify the infected sperm, resulting in CI when the modified sperm fertilizes an uninfected egg (Kose and Karr, 1995; Werren, 1997; Bossan et al., 2011). In the “toxin-antidote” model, CifB disrupts the processing of paternally derived chromosomes or nuclease activity and then changes or delays paternal chromatin condensation and separation during the first zygotic mitosis (Tram and Sullivan, 2002). Even so, much remains to be learned about the actual molecular mechanisms of CI induction and rescue, which can help account for CI in insects infected with different *Wolbachia* strains.

The feature of *Wolbachia* inducing a conditional sterility CI in infected insects is important for pest and disease control. In recent years, CI has been successfully explored to control the mosquito population and mosquito-borne diseases through population suppression or population replacement approaches. In the population suppression approach, large numbers of *Wolbachia*-infected male mosquitoes are released into the field, and the male sterility induced by CI causes significant drops in mosquito number. In the population replacement strategy, both *Wolbachia*-infected male mosquitoes and infected female mosquitoes are released, which can suppress mosquito-borne diseases by decreasing host virus transmission. Overall, *Wolbachia*-induced CI is central to both population suppression and population replacement programs (Ross et al., 2019).

### *Wolbachia*-induced pathogen inhibition

Reducing the infection or transmission of pathogens is another important property of *Wolbachia* used for pest and disease control. *Wolbachia* can inhibit RNA viral replication, which was initially discovered in *Drosophila melanogaster* (Hedges et al., 2008; Teixeira et al., 2008). Subsequently, *Wolbachia* were found to be broadly effective against mosquito-borne diseases such as Zika virus, chikungunya virus, dengue virus, yellow fever virus, and West Nile virus, making them less capable of transmitting infection to offspring and humans (Bian et al., 2013a; Ford et al., 2019). *Wolbachia* can also confer resistance against eukaryotic parasites (Bourtzis et al., 2014), providing a broad range of pathogen protection. Several studies have shown that the antiviral response is dramatically enhanced by *Wolbachia* newly transinfected to the host, although natural *Wolbachia*-infected mosquitoes are found to limit virus replication and transmission (Armbruster et al., 2003; Guo et al., 2022). Microinjection technology expands the entry of *Wolbachia* into new hosts. Once *Wolbachia* infects, *Wolbachia*-induced CI produces a frequency-dependent fitness advantage that can drive the spread of *Wolbachia* within new hosts (Sullivan, 2020). Data have further indicated that the extent of viral inhibition provided by transinfected *Wolbachia* depends on the *Wolbachia* variants, host species, virus and host-*Wolbachia*–virus interactions.

*Wolbachia*-induced pathogen inhibition is variable between related hosts and different *Wolbachia* strains in the same host, which is strongly linked to the density of *Wolbachia* in host tissues. In mosquitoes, virus inhibition correlates with higher *Wolbachia* density in the salivary glands, midgut, and ovaries. Unlike mosquitoes, high *Wolbachia* densities in the head, gut, and Malpighian tubules of *Drosophila* are thought to be important for virus inhibition (Osborne et al., 2012). It is widely believed that higher *Wolbachia* densities are important for effective antiviral behavior (Chrostek et al., 2013), and *Wolbachia* may confer virus inhibition by interfering with viral binding, entry into the cell, and RNA replication in the early stages (Schultz et al., 2018; Lu et al., 2020). Regardless of which pathway *Wolbachia* acts on, the production of progeny viruses from the same *Wolbachia*-infected cells is reduced, and virus dissemination and transmission are ultimately limited (Kaur et al., 2021). Interestingly, the varied extent of virus inhibition was also associated with viral dose. Recent data suggest that *w*Mel exhibits strong inhibition in high dengue dose mosquitoes, while inhibition appears lower or even increases virus transmission when the dengue dose is low (King et al., 2018).

**Figure 4:**
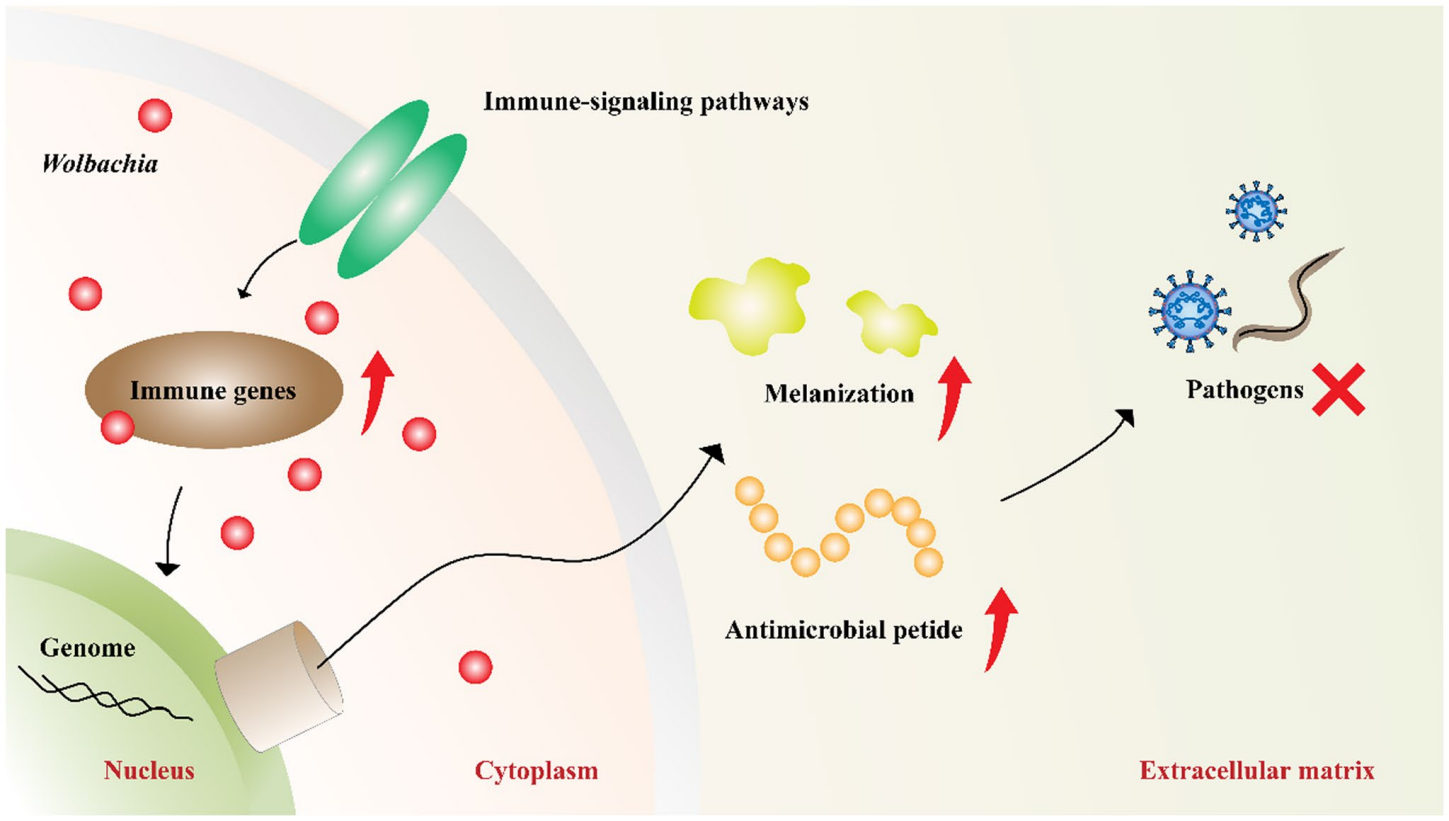
*Wolbachia* upregulate host innate immunity. *Wolbachia* enhance the synthesis of antimicrobial peptides and melanization by impacting the central genes of host immune-signaling pathways. The increased innate immunity partially accounts for *Wolbachia* inhibiting pathogens. Red dots: *Wolbachia*; red arrow: upregulation; red “X”: inhibition.

*Wolbachia*-induced pathogen inhibition may be related to the upregulation of host innate immunity (Figure 4). This is evident from the inhibition caused by *Wolbachia* newly transferred to hosts (Moreira et al., 2009; Walker et al., 2011; Ant et al., 2018). In mosquitoes with transinfected *Wolbachia* strains, *Wolbachia* upregulate the expression of genes involved in innate defense pathways and then prime insect innate immunity to block pathogen replication (Bian et al., 2013b; Moretti et al., 2018). However, inhibition associated with native *Wolbachia* variants does not show an immune-priming phenotype but does confer antiviral activity (Mousson et al., 2012). These results suggest that innate immune priming may occur in hosts with newly transinfected *Wolbachia* variants or novel host-*Wolbachia* associations (Rances et al., 2012).

**Figure 5:**
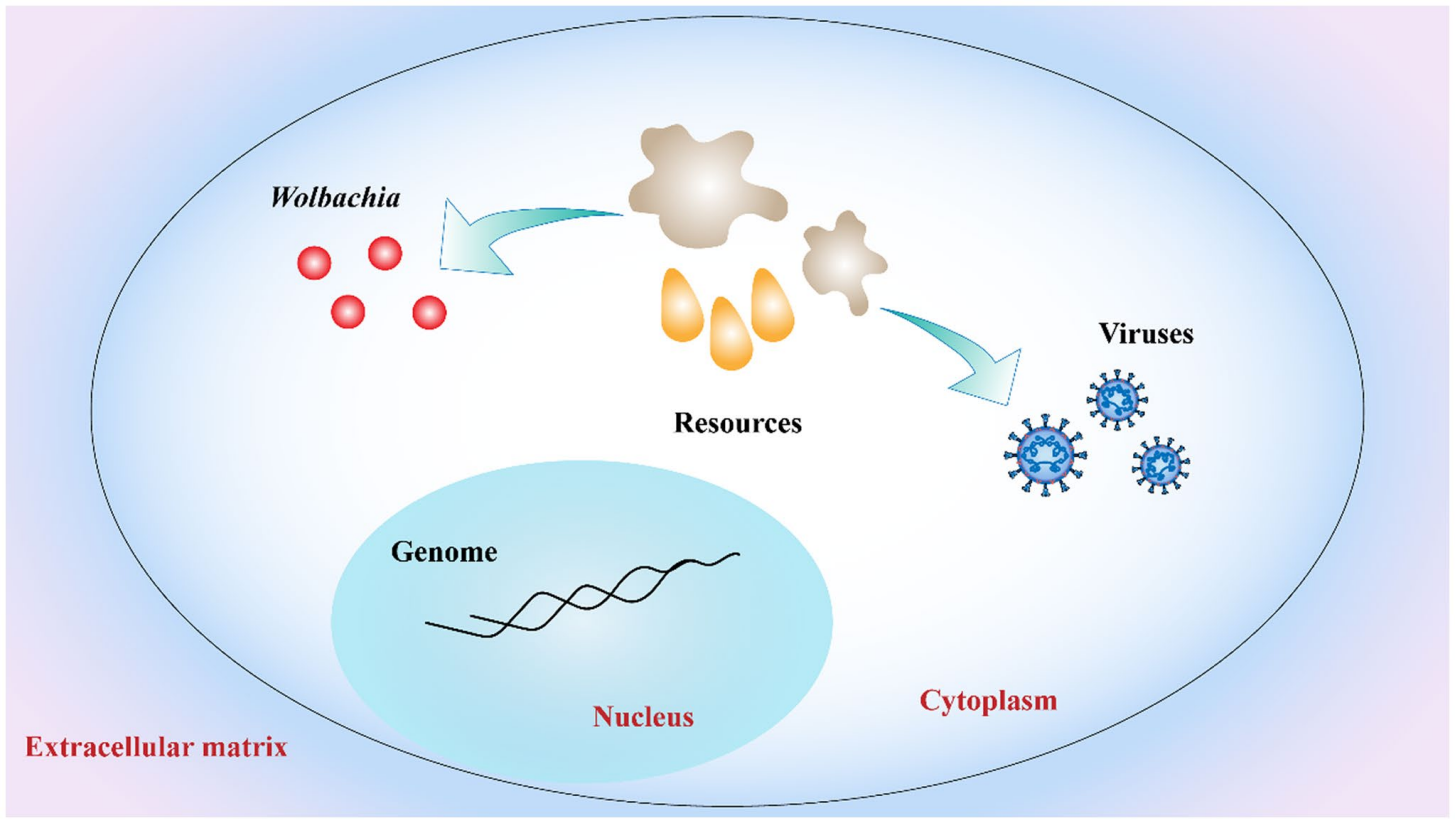
Competition for host cell resources. Both *Wolbachia* growth and virus replication rely on host cell resources. Resources depletion of host cells by *Wolbachia* interferes with virus replication in host limited cell resources. Red dots: *Wolbachia*; orange drop shapes and gray irregular shapes: host cell resources.

Another explanation for *Wolbachia* inhibiting virus replication is the competition for resources between viruses, *Wolbachia*, and the host cell (Figure 5). Viral replication and *Wolbachia* growth in the host are tightly regulated by cholesterol metabolism (Lin and Rikihisa, 2003). A recent study has shown that *Wolbachia* is unable to synthesize cholesterol *de novo* and that its replication is cholesterol dependent. Thus, cholesterol depletion of host cells by *Wolbachia* could directly interfere with virus replication in the same host (Rainey et al., 2014). In addition to cholesterol, iron homeostasis needs to be tightly regulated to enable viral replication and bacterial growth. In *Wolbachia*-infected mosquitoes, the iron-binding proteins transferrin and ferritin were upregulated, suggesting that *Wolbachia* regulated iron homeostasis (Kremer et al., 2009; Rances et al., 2012). However, this phenomenon is reversed when the host infect is infected with virus (Tchankouo-Nguetcheu et al., 2010). These experiments related to *Wolbachia*, antiviral activity and host cells are intriguing, clearly suggesting that host cell resources are important for both viral replication and *Wolbachia* growth. In summary, to develop alternative vector-control strategies, much remains to be learned concerning the mechanisms of *Wolbachia*-mediated pathogen inhibition.

## *Wolbachia* in planthoppers

### *Wolbachia* discovery in planthoppers

The small brown planthopper (*Laodelphax striatellus*; SBPH), brown planthopper (*Nilaparvata lugens*; BPH), and white-backed planthopper (*Sogatella furcifera*; WBPH) are the three serious and destructive pests of rice that directly cause 20 to 40% of crop loss globally each year (Yang and Zhang, 2016; Sullivan, 2020). *Wolbachia* in SBPH were first discovered in 1992 using partial sequences of the ribosomal DNA (Rousset et al., 1992), and WBPH was reported to harbor the same *Wolbachia* strain *w*Stri in 2003 (Kittayapong et al., 2003). In contrast to SBPH and WBPH, BPH was found to be infected with a different *Wolbachia* strain, *w*Lug. There were significantly different infection statuses among the three planthoppers (Table 1). The infection rate of *Wolbachia* in SBPHs increased gradually according to the investigation data from 1982 to 1994 (Noda, 1984a; Hoshizaki and Shimada, 1995). A recent study indicated that nearly all SBPHs were infected by *Wolbachia* in the rice-growing regions of China (Zhang et al., 2013). In WBPH, the infection rate of *Wolbachia* is different between females and males; nearly 100% of females are infected with *Wolbachia*, while only half of males are infected (Li et al., 2020). BPH is naturally infected by the *Wolbachia* strain *w*Lug at a prevalence of only ~18%, showing the lowest infection frequency among the three planthoppers (Qu et al., 2013).

**Table 1:** *Wolbachia* in small brown planthopper (SBPH), brown planthopper (BPH), and white-backed planthopper (WBPH).

	SPBH	BPH	WBPH
*Wolbachia* strain	*w*Stri	*w*Lug	*w*Stri
Infection frequency	100%	~18%	100% (female) ~50% (male)
Key features	Strong cytoplasmic incompatibility Maternal transmission Provide nutrients Increase fertility Increase resistance Protect against virus	Maternal transmission Provide nutrients Increase fertility Short lifespans Increase resistance	Weak cytoplasmic Incompatibility Maternal transmission Decrease fertility

*Wolbachia* is located in multiple tissues of planthoppers, including somatic tissues, ovaries, and testes. The somatic localization of *Wolbachia* is thought to facilitate their horizontal transmission, which also indicates the complex interactions between *Wolbachia* and the host. The reproductive localization of *Wolbachia* is thought to facilitate their vertical transmission. *Wolbachia* exhibit high-efficiency vertical transmission in planthoppers, as they do in the model insect *Drosophila* (Nakamura et al., 2012; Guo et al., 2018b), which occurs only in female hosts. In contrast to *Drosophila* ovarioles, planthopper ovarioles are of the telotrophic meroistic type and consist of a terminal filament, tropharium, and vitellarium (Szklarzewicz et al., 2013; Guo et al., 2018b). A cluster of nurse cells connected to the central trophic core radially arranged in the anterior of the tropharium; previtellogenesis arranged on the base of the tropharium (Szklarzewicz et al., 2007). Developing oocytes arrange in the vitellarium, which connects the tropharium through nutritive cords (Szklarzewicz et al., 2007, 2013). *Wolbachia* bind to Vg outside the ovarioles and endocytose into the tropharium of planthoppers during the early phase of vitellogenesis (Guo et al., 2018b). *Wolbachia* in the tropharium enter the arrested oocyte and establish an early infection as the trophic core divides. In addition, *Wolbachia* in the nurse cells spread into the developing oocytes through the nutritive cords that are wide channels formed between nurse cells and establish stable inheritance in host generation (Guo et al., 2018b). *Wolbachia* behavior during host embryogenesis is also well characterized. Microscopic observations indicated that *Wolbachia* were mainly localized at the anterior part cells of the embryo in early embryogenesis and then migrated to the posterior region during late embryogenesis, where gonads were formed (Guo et al., 2019). Research related to *Wolbachia* transmission in host oogenesis and embryogenesis can partially explain how *Wolbachia* exhibit high vertical transmission in planthoppers.

### *Wolbachia* functions in planthoppers

*Wolbachia* show different functions on three planthoppers (Table 1). Recent research has shown that *Wolbachia* provide beneficial effects to BPH. Egg production in *Wolbachia*-infected BPH females is higher than that in uninfected females. However, the longevity of *Wolbachia*-infected BPHs is shorter than that of uninfected BPHs, which may partially explain the high egg production and low prevalence of *Wolbachia* in wild BPH. Similar to BPH, *Wolbachia* also significantly increased the fecundity of SBPH, which may be associated with the high number of ovarioles that contain apoptotic nurse cells and mitotic germ cells (Guo et al., 2018b, 2020). In addition, *Wolbachia* affects the miRNA expression of SBPH to alter the expression of genes related to fecundity (Liu et al., 2019). Further experimental and genomic evidence demonstrated that *Wolbachia* increases the fecundity of BPH and SBPH females by synthesizing the essential nutrients biotin and riboflavin (Ju et al., 2020). In contrast, *Wolbachia* exhibit negative effects on WBPH; *Wolbachia*-infected females produce fewer eggs than *Wolbachia*-uninfected females (Li et al., 2022). Although many studies have focused on the interactions between *Wolbachia* and planthoppers, the mechanism of *Wolbachia*-mediated alterations in planthopper oogenesis has not yet been explored.

The CI phenotype in laboratory and wild SBPH populations was found in 1984 (Noda, 1984b). In 1992, the CI phenotype in SBPH was confirmed, which was caused by *Wolbachia w*Stri (Rousset et al., 1992). *w*Stri induced strong CI in SBPH, and the level of CI remained high regardless of the age of *Wolbachia*-infected males. There are no viable eggs from *Wolbachia*-infected SBPH females that mated with uninfected SBPH males. RNA-seq comparative analysis of *Wolbachia*-infected and uninfected SBPH shows that iLvE mediates branched-chain amino acid biosynthesis and may be associated with *Wolbachia*-induced CI (Ju et al., 2017). Knocking down iLvE expression in *Wolbachia*-uninfected SBPH males partially rescued fertility in crosses between these males and *Wolbachia*-infected females. Wild WBPH populations are infected by the same *Wolbachia w*Stri as SBPH are, while the level of CI in WBPH is very weak or even zero. However, a strong CI phenotype was expressed when WBPH was double-infected with *Wolbachia* and *Cardinium* bacterium, indicating that *Wolbachia* may only play an auxiliary role in the CI of WBPH (Li et al., 2022). Interestingly, *Wolbachia w*Lug in wild BPH populations lacks the ability to induce CI. A recent study showed that BPH infected with *w*Stri by microinjection exhibited a high CI level, although the CI level was much lower than that in the original host SBPH (Gong et al., 2020).

In recent years, effects other than reproductive effects on planthoppers have received increasing attention. Studies have shown that *Wolbachia* of planthoppers increase resistance to insecticides, protect against some RNA viruses, and have other effects. In SBPH, *Wolbachia w*Stri is associated with increased resistance to the insecticide buprofezin, although there is no relationship between *Wolbachia* density and resistance (Li et al., 2020). BPH increased insecticide susceptibility and decreased detoxification metabolism when the density of *Wolbachia* was decreased by high temperature (Zhang et al., 2021). Further results indicated that *w*Lug orchestrates the detoxification metabolism of BPH via the CncC pathway to promote host insecticide resistance. In addition, *Wolbachia* wStri was recently shown to inhibit the growth of positive-sense RNA mosquito viruses, and the inhibition level was up to 99.9%. The presence of *w*Stri did not affect the growth of the negative-sense RNA viruses in the *Bunyaviridae* and *Rhabdoviridae* families (Schultz et al., 2018). *w*Stri in *Aades albopictus* cells has also been shown to repress ZIKV, and the inhibited stages of the ZIKV life cycle were identified to two distinct blocks, including reduction of ZIKV entry into cells and distraction viral genome replication in *Wolbachia*-infected cells. The addition of a cholesterol-lipid supplement partially rescued ZIKV entry in *w*Stri-infected cells but did not rescue viral replication, showing that viral entry is affected in a cholesterol-dependent manner (Schultz et al., 2018). *Wolbachia w*Stri has the ability to inhibit a wider variety of positive-sense RNA viruses, making it an attractive candidate for future vector-controlled approaches to limit viral infection and spread.

## Opportunities and challenges

*Wolbachia*-based mosquito control strategies have been shown to be effective at limiting arbovirus disease spread in approximately 23 countries (Gong et al., 2023). Among them, over 8 countries have used *Wolbachia*-based mosquito population suppression strategies, which closely depend on *Wolbachia*-induced CI. The most important aspect of this strategy is that stable and heritable CI-induced *Wolbachia* infections should be established in target species. To control mosquitoes, adult sterile males with artificial *Wolbachia* infection have been released to mate with wild females. The eggs produced by these females are perishable, resulting in a target species population decline in a given period. However, the mass release of adult sterile males involves a potential risk of accidentally releasing fertile CI-induced *Wolbachia*-infected females. Insects are traditionally sterilized by radiation; combining *Wolbachia*-induced CI with the radiation sterilization technique can sterilize any residual females that are not removed from the released males using low-dose irradiation. Recent field trials indicated that the combination of *Wolbachia* and radiation sterilization resulted in a near elimination of mosquito populations (Zheng et al., 2019). Another efficient vector-control strategy is *Wolbachia*-based population replacement, which has been successfully used in 15 countries. The success of this strategy relies on two aspects of *Wolbachia*: pathogen inhibition and CI drive. Rather than releasing large numbers of *Wolbachia*-infected males to suppress insect populations, *Wolbachia*-infected females would be released to replace a wild uninfected population by a CI-based drive, reducing their vector competence and inhibiting arboviral disease (Kaur et al., 2021).

In recent years, great progress has been made in developing possible applications for protecting plants from planthoppers and their associated diseases. BPH is naturally infected with *Wolbachia* strain *w*Lug at a low prevalence that does not cause CI. Gong et al. (2020) established a *w*Stri-infected BPH line by withdrawing the embryo cytoplasm of SBPH and injecting it into the embryos of BPH. *w*Stri maintained perfect maternal transmission in the new host BPH. The *w*Stri-infected BPH exhibited near 100% CI, although it was slightly lower than that in its native host SBPH (Gong et al., 2020). The high level of CI and low fitness costs of *w*Stri-infected BPHs enable individuals infected with *w*Stri to rapidly invade BPH populations. Furthermore, *w*Stri-infected BPH dramatically reduced planthopper RRSV viral loads and viral transmission to rice plants. The viral load in *w*Stri-infected BPH decreased 75% relative to that in uninfected BPH (Gong et al., 2020). Otherwise, rice seedlings attacked by *w*Stri-infected BPH resulted in a dramatic 82% lower incidence of viral infection compared with that attacked by uninfected BPH (Gong et al., 2020). Above all, the *w*Stri strain appears to be well suited for the *Wolbachia*-based replacement strategy to control BPHs and their associated diseases, although much work still needs to be done before strategy implementation.

**Figure 6:**
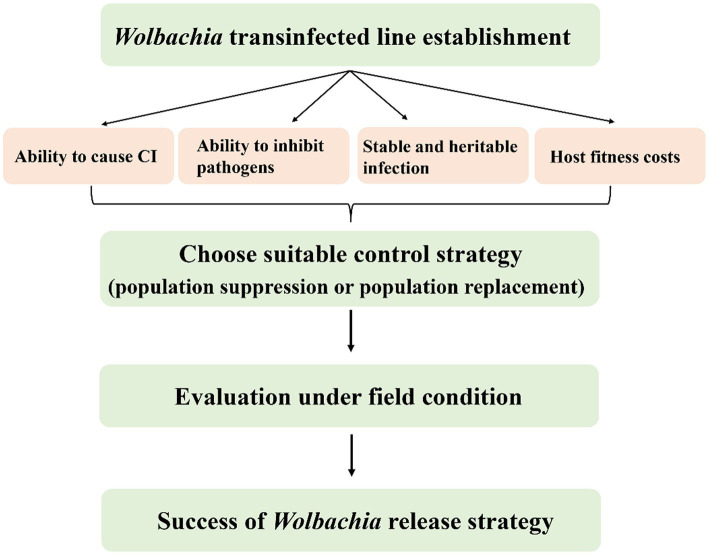
Properties of *Wolbachia* used for planthoppers control. The success of *Wolbachia*-based population control strategies relies on the important properties of *Wolbachia* including host reproduction alteration, pathogen inhibition, stability transmission and weak or no host fitness cost. CI, cytoplasmic incompatibility.

Possible *Wolbachia*-based population control applications for SBPH and WBPH are more complex than those for in BPH. SBPH and WBPH naturally carry *Wolbachia*, so double infections are needed for population replacement, whereas double infected strains or novel strains with native *Wolbachia* removed but carrying another variant added are needed for population suppression. First, a stable and heritable CI-induced *Wolbachia* infection line should be established by artificial transfection (Figure 6). Although embryonic microinjection technology significantly promotes *Wolbachia* transfection efficiency from donors to recipients, many problems remain, such as selecting useful *Wolbachia* variants, which have desirable phenotypic effects for alternative strategies and maintain stability in the longer term. Apart from native *Wolbachia*, new *Wolbachia* interactions with other endosymbionts and the complex microbiome could influence host fitness and indirectly affect *Wolbachia* invasion (Ross et al., 2019).

Host fitness cost is an important determinant in *Wolbachia*-based pest control strategies. The fitness of hosts is altered when hosts are infected with different *Wolbachia* strains. In general, natural *Wolbachia* infections are benign or even beneficial to the host, such as increasing fertility or lifespans as well as inhibiting the virus. In contrast, diverse negative effects on fitness are found when *Wolbachia* are transferred to novel hosts, depending on the *Wolbachia* strain and host. It is usually difficult to predict the fitness effects of *Wolbachia* on novel hosts because *Wolbachia* densities and tissue distributions dramatically change from native to novel hosts. Most negative effects are that *Wolbachia* transfections often reduce novel host fecundity or egg hatch, which may prevent transinfected *Wolbachia* establishment if they are too severe. The overall impact of *Wolbachia* infections on host fitness is often insufficiently estimated because it strongly depends on the environmental context. The fitness effects of *Wolbachia* observed in standard laboratory studies are only partial to estimate the dynamics of *Wolbachia* in natural populations.

Choose suitable *Wolbachia*-based population control strategies, population suppression or population replacement, which closely depend on the biology of the target pest. In mosquitoes, both males and females can feed on damaged and intact vegetative tissue, plant juices, damaged fruits, and homopterans, which act as an energy source for their physiological maintenance and locomotion. Only female mosquitoes bite animals or humans to take a blood meal, which is required for egg development. Therefore, there is no or little threat to animals or humans using *Wolbachia*-based mosquito suppression strategies by releasing adult sterile males. To efficiently reduce the prevalence of mosquito-borne diseases, *Wolbachia*-based replacement strategies were carried out by the release of *Wolbachia*-transinfected antiviral females or eggs. Nevertheless, both female and male planthoppers suck rice sap and transmit viral diseases, and there is no mature biotechnology or equipment for sex sorting to date. Hence, a population replacement strategy may be more suitable for controlling planthoppers than population suppression based on the current knowledge of interactions between *Wolbachia* and the host (Table 2). Moreover, the chosen strategies should be evaluated under field conditions to demonstrate the possibility of their practical implementation in the future.

**Table 2:** *Wolbachia*-mediated mosquito control strategies and the possible strategy for planthoppers.

	Mosquitoes	Planthoppers
Native *Wolbachia*	Yes/No	Yes/No
*Wolbachia* transfection	Yes	Yes
Key features	Cytoplasmic incompatibility Maternal transmission Pathogen inhibition	Cytoplasmic incompatibility Maternal transmission Pathogen inhibition
Host fitness cost	No/Weak	Weak
Key biology of host	Females bite human and transmit virus Males feed plant juices	Both females and males destruct rice and transmit virus
Control strategies	Population suppression/population replacement	Population replacement

## Author contributions

YG and YL wrote the manuscript. JS and YW performed the images processing. All authors contributed to the article and approved the submitted version.

## Funding

This study was supported by the Research and Development program in key areas of Guangdong Province (Grant No. 2021B0707010010), the Special Fund for Scientific Innovation Strategy-Construction of High-Level Academy of Agriculture Science (Grant Nos. R2021YJ-YB2007 and 202117TD), and the National Natural Science Foundation of China (grant no. 31901879).

## Conflict of interest

The authors declare that the research was conducted in the absence of any commercial or financial relationships that could be construed as a potential conflict of interest.

## Publisher’s note

All claims expressed in this article are solely those of the authors and do not necessarily represent those of their affiliated organizations, or those of the publisher, the editors and the reviewers. Any product that may be evaluated in this article, or claim that may be made by its manufacturer, is not guaranteed or endorsed by the publisher.
